# Occupational stress and musculoskeletal disorders in firefighters: the mediating effect of depression and job burnout

**DOI:** 10.1038/s41598-024-55468-w

**Published:** 2024-02-26

**Authors:** Amir Hossein Khoshakhlagh, Saleh Al Sulaie, Marziyeh Mirzahosseininejad, Saeid Yazdanirad, Robin Marc Orr, Fereydoon Laal, Umesh Bamel

**Affiliations:** 1 Department of Occupational Health, School of Health, Kashan University of Medical Sciences, Kashan, Iran; 2 Department of Mechanical and Industrial Engineering, College of Engineering and Computers in Al-Qunfudah, Umm Al-Qura University, 21955 Makkah, Saudi Arabia; 3 Fire Safety Section of Department of Health, Safety and Environment of Sarcheshmeh Copper Complex, National Iranian Copper Industries Co, Rafsanjan, Kerman Iran; 4 Social Determinants of Health Research Center, Shahrekord University of Medical Sciences, Shahrekord, Iran; 5 Tactical Research Unit, Bond University, Gold Coast, Australia; 6 Department of Occupational Health Engineering, Social Determinants of Health Research Center, Birjand University of Medical Sciences, Birjand, Iran; 7 OB & HRM Group, International Management Institute New Delhi, New Delhi, India

**Keywords:** Job stress, PTSD, Work-related musculoskeletal disorders, Firefighters, Depression, Burnout, Health occupations, Risk factors

## Abstract

The firefighting profession carries a heightened risk of musculoskeletal disorders. A firefighter’s job is physically demanding and includes activities such as running, climbing, dragging, and lifting. Often, these tasks are unpredictable, performed in harsh environments, and have been found to cause psychological stress. The purpose of this study was to investigate the effects of occupational stress on work-related musculoskeletal disorders (WRMSD) in firefighters. In addition, the mediating effects of depression and job burnout on proposed relationships were examined. Data informing this study were collected using a survey questionnaire. The survey questionnaire included the Beck Depression Inventory, the Center for Epidemiological Studies Depression Scale (CES-D), the Maslach Burnout Inventory, the Post Traumatic Stress Disorder Inventory (PCL), and the Nordic Musculoskeletal Questionnaire. Collected data were analyzed using structural equation modeling approach in AMOS. The results of the 2339 responding firefighters suggest that work related stress is positively related to WRMSDs in firefighters and can lead to musculoskeletal symptoms through four paths, being emotional exhaustion, personal accomplishment, CES-D total score, and depersonalization. Through depersonalization, job stress had the most significant impact on musculoskeletal symptoms (coefficient = 0.053). Furthermore, the results showed that post-traumatic stress disorders (PTSD) can affect musculoskeletal symptoms through ten paths, again through depersonalization, PTSD had the most significant impact on musculoskeletal symptoms (coefficient = 0.141). The results of this study suggest that organizations should design interventions and policies to prevent and manage occupational stress, depression, and job burnout to negate its undesired consequences on firefighters’ health (i.e. WRMSD).

## Introduction

Work-related musculoskeletal disorders (WRMSDs) are common occupational health issues, often involving multiple factors^1–3^. WRMSDs include disorders of muscles, tendons, peripheral nerves, joints, bones, ligaments, and blood vessels, resulting from repetitive stress over time or acute (immediate) trauma and presenting with symptoms including discomfort, pain, fatigue, dryness, swelling, limited range of motion, muscle stiffness, numbness, or tingling^4^. According to the World Health Organization^3^, WRMSDs are the second largest occupational disease, after respiratory diseases^3^. A WHO International Labour Organization (WHO-ILO) global monitoring report on ‘work-related burden of diseases and injury’ reported occupational ergonomic factors in the top three risk factors for causing worker diseases and injury along with long working hours and exposure to particulate matters^5^. According to surveys conducted in Europe in 2005, 23 to 25% of workers from 27 different regions of the European Union reported WRMSDs^4^. A similar study in Greece reported the prevalence rate of these disorders to be 46–47%^6–8^. European Union Occupational Safety and Health Administration (EU-OSHA) reports of 2020 suggest that around 60% of all workers with workplace related health issues reported WRMSD as a serious health problem^9^. Every year in the United States (US), more than one million workers suffer from musculoskeletal injuries, especially low back injuries^10^. These injuries were found to cause 30% of total disability and 40% of partial disability in the US^10^. Further, an American Statistics Organization report published in 2014 stated that approximately 32% of all occupational diseases are attributed to musculoskeletal disorders^11^.

There is a high degree of correlation between WRMSDs and workers’ disability and injury, loss of work time, poor productivity, increased costs, and economic losses^12–14^. In addition to workers’ health and productivity loss, WRMSDS are hurting organizations economically as 40% of work-related compensation costs are related to WRMSDs^15,16^. Further, WRMSDs were the main source of disability and related costs. In 2001, more than 1.13% of the general budget of the Iranian government was spent on these disorders^17^. Based on the available statistics, nearly 48% of work-related illnesses are accumulated injuries that are caused by physical or mechanical factors^18^.

Firefighters, due to the physical demands of their work, are susceptible to WRMSDs. Despite their vulnerability, there is limited literature focusing on this group. The existing research on firefighters indicates that back pain and knee pain are prevalent and often occurring during firefighting activities (38.3%), providing emergency services (37.7%), conducting rescue operations (12.4%), and during execution of other related tasks^19^. Negm et al.^20^ corroborate and confirm the prevalence of WRMSDs in firefighters’ neck (20%), back (33%), arm (44%), and forearm (45%). Likewise, Aurangabadkar et al.^19^ identified various complaints among firefighters, with prevalence rates of 24% for neck issues, 23% for shoulder problems, 13% for elbow discomfort, 7% for upper back concerns, and 6% for lower back complaints. This volume of evidence demonstrate that firefighters suffer from WRMSDs. The nature of firefighters’ job is often demanding, and they usually respond to emergency situations such as fire suppression, rescue operations, handling of hazardous elements, and so on^21^. The work of a firefighter is often unpredictable, and physically and mentally challenging. Thus firefighters may experience a considerably high degree of occupational stress^22^.

The results of some studies show that two important factors of PTSD and job stress can affect musculoskeletal disorders. Jahakhan et al. investigated the role of PTSD in the development of chronic musculoskeletal pain and disability and concluded that there is an association between PTSS and the development of longer-term pain and disability^23^. The results of a study performed by Ouimette et al. showed that PTSD diagnosis and symptoms were associated with a higher likelihood of musculoskeletal disorders^24^. Shaygan and Yazdanpanah concluded that job stress can influence chronic pain with a direct effect coefficient of 0.477^25^. Certainly, this evidence suggests that PTSD and occupational stress can be notable risk factors for WRMSDs. In addition to the direct effect of PTSD and job stress on musculoskeletal disorders, some variables such as depression and job burnout may mediate these relations. The results of some research explain it. The results of a study performed by Ng et al. showed that depression can affect musculoskeletal disorders with an effect coefficient of 0.28^26^. Langballe et al. performed a study on the relationship between burnout and musculoskeletal pain using structural equation modeling and concluded that the burnout dimensions can impress on musculoskeletal pain with the effect coefficients between and 0.17 and 0.64^27^. On the other hand, based on the results of the study, PTSD and job stress are related to depression and burnout. The results of a study conducted by Ying et al. showed that there are longitudinal associations among PTSD symptoms, depressive symptoms, and burnout in the different age groups^28^. Peng et al. concluded that PTSD can affect pain severity through depression with an indirect effect of 0.284^29^. Also, the results of a study performed by Shaygan and Yazdanpanah revealed that job stress can indirectly influence chronic pain through depression with an indirect effect coefficient of 0.42^25^.

Based on the results of the mentioned studies, the assumptions and aims of the present study were written and the theoretical model in structural equation modeling (SEM) was drawn. Firstly, it was hypothesized that PTSD and job stress can directly affect musculoskeletal disorders among firefighters. Moreover, secondly, it was hypothesized that PTSD and job stress would influence burnout dimensions and depression and through these factors affect musculoskeletal symptoms indirectly. Also, while proposing this, present study built on evidence that^30^ suggest role of PTSD in triggering the risk of other health disorders. Considering the stated assumptions, the present study aimed to investigate the role of PTSD and job stress on WRMSDs directly and indirectly through depression and three dimensions of burnout (emotional exhaustion, depersonalization, and personal accomplishment) using SEM.

## Materials and methods

### Participants

This cross-sectional study was conducted in 2022 with participants recruited from 131 fire stations in Northern Iran via random sampling during this time. For selecting the samples, a list of the circa 5000 firefighters serving in the fire stations was prepared. The Cochran formula^31^ was used to compute the sample size whereby, based on a population size of 5000 firefighters, the confidence level, margin error, and population proportion considered as 99%, 2%, and 50%, respectively, a sample size of 2022 firefighters was needed. As such, and to allow for drop off, 3000 participants were randomly selected for the list of 5000 through assigning a number code to each person. Once the list was generated, the medical records of these individuals were studied. In Iran, the mental and physical health of firefighters is examined annually and is recorded in their medical records. Information on eligibility criteria were evaluated through the review of medical records of individuals. Following that, 2834 firefighters with inclusion criteria were invited to the study. Then, the firefighters were invited to participate in the study through phone call. Among them, 2617 firefighters accepted to participate in the study. Finally, out of 2617 responders in the study, 2339 firefighters filled out the questionnaires accordingly. The response rate was 89.38 percent. The inclusion criteria were: (a) age ranged from 18 to 60 years, (b) having worked as a firefighter for more than one year, (c) not having diagnosed mental disorders, and (d) not taking any psychiatric drugs. Exclusion criteria included: (a) having a history of major trauma (such as driving, sports, and occupational accidents), rheumatic diseases, spine surgeries, and large joint surgeries, and having musculoskeletal structural deformities (such as spinal abnormalities and genu varum); a history of long-term intake of corticosteroids and immunosuppressants; and (c) non-cooperation during the study. Also, participants who did not fill the questionnaire or filled it incompletely were excluded from the study. The protocol of the study was reviewed and approved by the medical ethics committee of Birjand University of Medical Sciences. All steps of the study were in accordance with the ethical code IR.BUMS.REC.1401.195. All participants consciously consented to participate via a consent form provided by the ethics committee.

Given that the samples were in various fire stations, the data were collected from August to November in 2022. The researchers went to the workplaces of the participants where, before data collection, a general description of the study was provided during their rest time. Following this, paper-based questionnaires were given to the participants and they were asked to complete them. If the participants had any doubts or questions, the researchers were available to answer them. Following completion of the questionnaires, all copies were collected by the researchers. The questionnaires comprised of demographic information, the Occupational Stress Questionnaire-HSE, the PTSD Checklist, the Maslach Burnout Inventory (MBI), the Center for Epidemiologic Studies Depression Scale (CES-D), and the Nordic Musculoskeletal Questionnaire (NMQ). Further information on these surveys is provided below.

### Outcome measures

#### Demographic information questionnaire

The demographic information questionnaire included several questions through which to descriptively profile the participants and included age, education, marital status, body mass index, second job status, and smoking status.

#### Occupational stress questionnaire-HSE

The Health and Safety Executive Management Standards Indicator Tool (HSE-MS IT) is a 35-question questionnaire designed to evaluate psychological variables related to the evaluation of exposure to stressful factors, in accordance with the management standards developed in England^32^. This questionnaire consists of seven dimensions: demands (8 items; a sample item: ‘Different groups at work demand things from me that are hard to combine’), control (6 items; a sample item: ‘I can decide when to take a break’), officials’ support (5 items; a sample item: ‘I am given supportive feedback on the work I do’), colleagues’ support (4 items; a sample item: ‘If work gets difficult, my colleagues will help me’), relationships (4 items; a sample item: ‘I am subject to personal harassment in the form of unkind words or behavior’), role (5 items; a sample item: ‘I am clear what is expected of me at work’), and changes (3 items; a sample item: ‘I have sufficient opportunities to question managers about change at work’). The scoring of this tool is based on a Likert-type scale ranging from 1 (never) to 5 (always), and its final cumulative score ranges from 35 to 175. The scores of some questions are reversed. Higher scores on the HSE-MS IT scale indicate lower risk of stress. Marcatto et al.^33^ investigated the validity of this questionnaire and concluded that HSE-MS IT had the validity and specific sensitivity to assess different aspects of work-related distress.

#### Post traumatic stress disorder inventory

This questionnaire was developed by Weathers et al.^34^ in 1991 based on the DSM diagnostic criteria for the US National PTSD Center. It contains 17 items. This questionnaire has four dimensions including intrusive cluster (5 items; a sample item: ‘Have you had painful images, memories or thoughts of the events?’), avoidance cluster (2 items; a sample item: ‘Have you been avoiding any thoughts or feelings about the event?’), amnesia and numbing cluster (5 items; a sample item: ‘Have you found yourself unable to recall important parts of the event?’), and hyperarousal cluster (5 items; a sample item: ‘Have you had trouble falling asleep or staying asleep?’). The subjects answer each question with one of two options: ‘yes’ (1 point) or ‘no’ (0 point). Higher scores on this scale suggest a higher risk of PTSD. Davidson et al.^35^ evaluated the validity and reliability of this questionnaire and concluded that this tool had good reliability and validity.

#### Maslach burnout inventory

This MBI was developed by Maslach (1981) and is used to estimate burnout^36^. The questionnaire contains 22 items to measure emotional exhaustion (9 items; a sample item: ‘I feel emotionally exhausted because of my work.’), depersonalization (5 items; a sample item: ‘I get the feeling that I treat some clients/colleagues impersonally, as if they were objects.’), and personal accomplishment (8 items; a sample item: ‘I can easily understand how my recipients feel about things.’) in the framework of professional activity. Item scoring for this inventory is based on a 7-point Likert scale from 0 (‘never’) to 6 (‘daily’)^37^. Shamloo et al.^38^ studied the validity and reliability of this questionnaire and concluded that this tool has the acceptable validity and reliability. In their study the parameter total correlation and internal consistency (total alpha) were 0.79, 0.85, and 0.87, accordingly.

#### Center for epidemiologic studies depression scale (CES-D)

This CES-D was created by Radloff in 1977 to evaluate depression in the general population^39^. While this scale is available in versions of 4, 10, and 20 questions, in this study, the full version with 20 questions was used. A sample item of this tool is: ‘I was bothered by things that usually do not bother me.’ Each question is scored on a 4-point Likert scale from 0 to 3 points (0 = rarely or none of the time, 1 = some or little of the time, 2 = moderately or much of the time, 3 = most or almost all the time), and the total score of this tool is between 0 and 60^39^. A higher score means a higher level of depression. Fountoulakis et al.^40^ determined the sensitivity and specificity of the scale exceeded 90.00 at 23/24, with a reliability coefficient for the scale scoring a Cronbach’s Alpha of α = 0.95. In the same study, test–retest reliability was found to range 0.45–0.95^40^.

#### Nordic musculoskeletal questionnaire (NMQ)

The NMQ evaluates pain in the neck, shoulder, elbow, wrist, back, waist, thigh, knee, and leg. This questionnaire was designed by Kuorinka et al. in 1987 and is now known as the NMQ^41^. This questionnaire has two parts: the first part contains general questions such as work history, weight, and height, and the second part contains questions related to the complication and discomfort in nine body regions (neck, shoulders, elbows, hands, back, waist, thighs, knees, and feet) during the last year. A ‘yes’ (1 point) or ‘no’ (0 point) response is given by the respondent for each body region. Chairani^42^ evaluated the validity and reliability of this tool and concluded that it can clarify workers who have real pain in the various body regions.

### Data analysis

**Figure 1:**
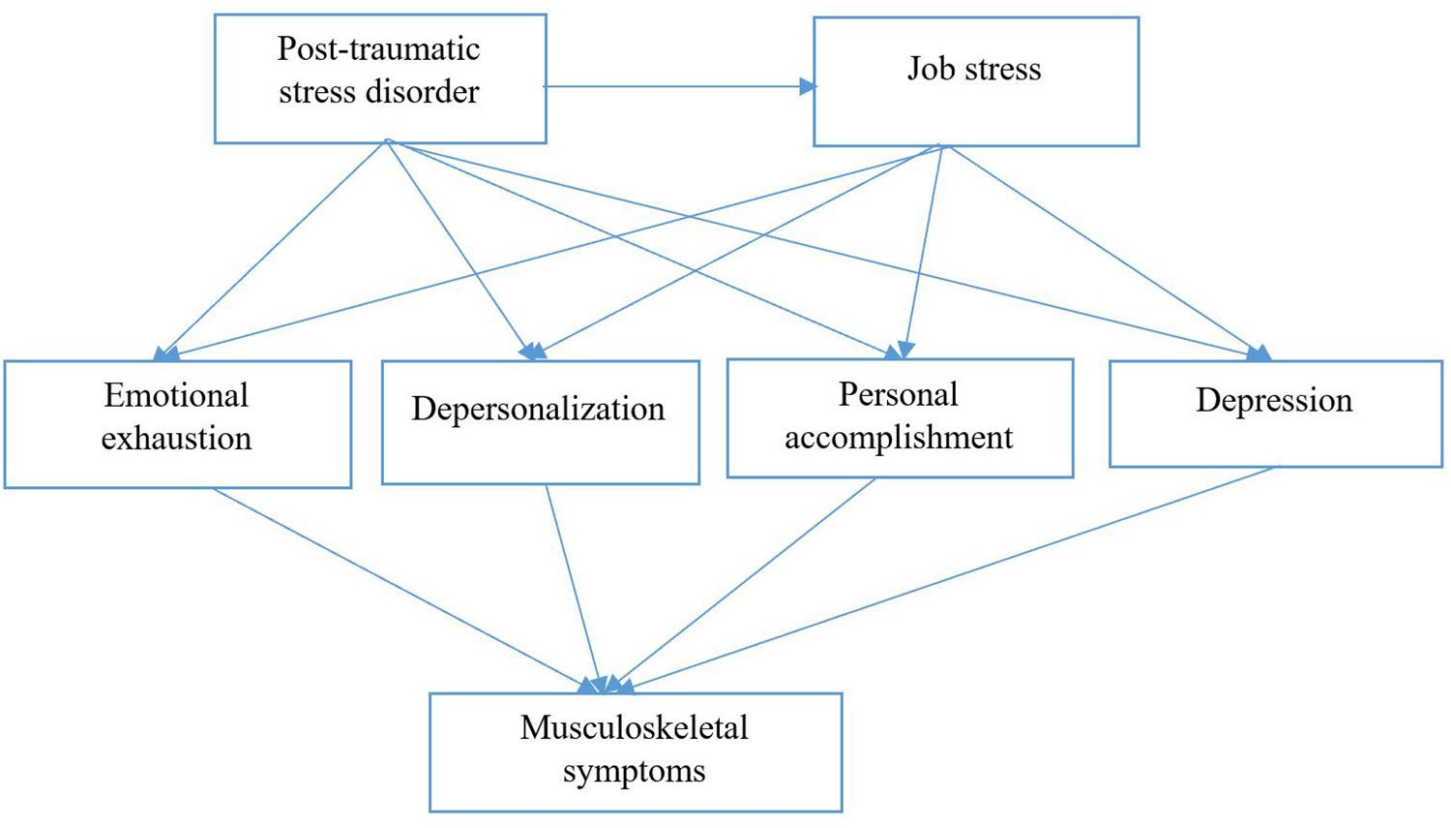
Theoretical model of the study.

Data were analyzed by the statistical package for the social sciences (SPSS) software version 26 (IBM Corporation, Armonk, NY, USA). The normality of variables was examined by skewness and kurtosis curves. Given that the statistical distribution of all parameters was normal, the correlation coefficients were computed by Pearson’s Correlations. Following this, statistical modeling using a theoretical model, structural equation modeling (SEM) was generated in AMOS software version 26 (IBM Corporation, Armonk, NY, USA). SEM involves a model representing how various parameters are thought to causally connect to one another. Figure 1 depicts the conceptual model of this study.

### Ethics approval and consent to participate

Written consent was taken from all participants to participate in this study. The Research Ethics Committee of Birjand University of Medical Sciences (BUMS) granted ethics approval for this study (No. IR.BUMS.REC.1401.195). Also, we confirm that all methods were performed in accordance with the relevant guidelines and regulations.

## Results

Of the initial 3000 selected firefighters, 2834 meet the study eligibility criteria and were invited to participate in the study. Among them, 2617 firefighters volunteered. Of these 2617 firefighters, 2339 firefighters filled out the questionnaires accordingly. The response rate was therefore calculated as 89.38%. The mean (± standard deviation) values for age and body mass index were 32.30 (± 5.74) years and 26.64 (± 6.39) kg/m^2^, respectively. Table 1 summarizes the descriptive statistics related to demographic variables. Based on the results, most of the participants (55.1%) were within 30 to 40 years of age, with an education degree at a level of associate degree (37.1%), with a body-mass index of 18.5 to 25 (42.8%), without a second job (55.8%), a married status (66.9%), and a non-smoker (77.9%). Table 2 describes the descriptive information of the studied variables. The score of the musculoskeletal symptoms also had a wide range from 0 to 9.

**Table 1:** Descriptive statistics related to demographic variables.

Demographic variables		Frequency	Valid percent (%)
Age (years)	Lower than 30	908	38.8
	30–40	1288	55.1
	41–50	135	5.8
	Higher than 50	8	0.3
Education degree	Under diploma	555	23.7
	Diploma	766	32.7
	Associate degree	867	37.1
	Bachelor’s degree	149	6.4
	Master’s degree and higher	2	0.1
Body mass index (kg/m^2^)	Lower than 18.5	78	3.3
	18.5–24.9	1001	42.8
	25–30	864	36.9
	Higher than 30	396	16.9
Second job	No	1305	55.8
	Yes	1034	44.2
Marital status	Single	775	33.1
	Married	1564	66.9
Smoking	Yes	516	22.1
	No	1823	77.9

**Table 2:** The descriptive information of the studied variables.

Studied variable		Range	Mean	Std. Deviation	Interpretations
Job stress		35.00–175.00	109.50	22.79	Higher value = lower job stress
Post-traumatic stress disorder		0.00–17.00	6.14	2.89	Higher value = higher PTSD
Burnout	Emotional exhaustion	0.00–54.00	22.29	11.82	Higher value = higher burnout
	Depersonalization	0.00–30.00	11.34	6.37	Higher value = higher burnout
	Personal accomplishment	0.00–48.00	26.23	8.18	Higher value = lower burnout
Depression		0.00–60.00	14.43	10.23	Higher value = higher depression
Musculoskeletal symptoms		0.00–9.00	3.73	2.91	Higher value = higher musculoskeletal symptoms

Table 3 describes the correlation coefficients of all variables. The results indicated that there were significant correlations among all variables and musculoskeletal symptoms (*p* < 0.01). Of the job stress dimensions, demands (r = − 0.659, *p* < 0.01) had the highest correlation with musculoskeletal symptoms. Among the burnout dimensions, the greatest correlation coefficient with musculoskeletal symptoms was related to the variable of the depersonalization (r = 0.727, *p* < 0.01). Among all variables, the highest correlation coefficient was between post-traumatic stress disorder and musculoskeletal symptoms (r = 0.820, *p* < 0.01).

**Table 3:** Correlation matrix of the studied variables.

Variable		1	2	3	4	5	6	7	8	9	10	11	12	13	14
1	Role	0.842													
2	Relationships	0.24**	0.911												
3	Managerial support	0.41**	0.41**	0.922											
4	Peer support	0.67**	0.40**	0.52**	0.897										
5	Control	0.68**	0.30**	0.49**	0.69**	0.933									
6	Demands	0.37**	0.69**	0.49**	0.46**	0.41**	0.878								
7	Changes	0.35**	0.48**	0.58**	0.49**	0.48**	0.51**	0.902							
8	Overall job stress	0.75**	0.67**	0.73**	0.81**	0.79**	0.77**	0.702**	0.911						
9	Post-traumatic stress disorder	− 0.39**	− 0.69**	− 0.45**	− 0.50**	− 0.42**	− 0.68**	− 0.45**	− 0.69**	0.966					
10	Emotional exhaustion	− 0.34**	− 0.62**	− 0.43**	− 0.43**	− 0.38**	− 0.61**	− 0.43**	− 0.63**	0.76**	0.902				
11	Depersonalization	− 0.38**	− 0.63**	− 0.43**	− 0.46**	− 0.403**	− 0.62**	− 0.45**	− 0.65**	0.74**	0.81**	0.831			
12	Personal accomplishment	0.002	0.33**	0.26**	0.14**	0.10**	0.30**	0.28**	0.26**	− 0.47**	− 0.46**	− 0.45**	0.745		
13	Depression	− 0.36**	− 0.54**	− 0.33**	− 0.45**	− 0.38**	− 0.48**	− 0.39**	− 0.56**	0.67**	0.57**	0.54**	− 0.28**	0.879	
14	Musculoskeletal symptoms	− 0.47**	− 0.64**	− 0.51**	− 0.56**	− 0.49**	− 0.66**	− 0.50**	− 0.74**	0.82**	0.71**	0.73**	− 0.45**	0.59**	0.941

The diagonal of the table reports reliabilities of the variable.

*p < 0.05: **p < 0.01.

**Figure 2:**
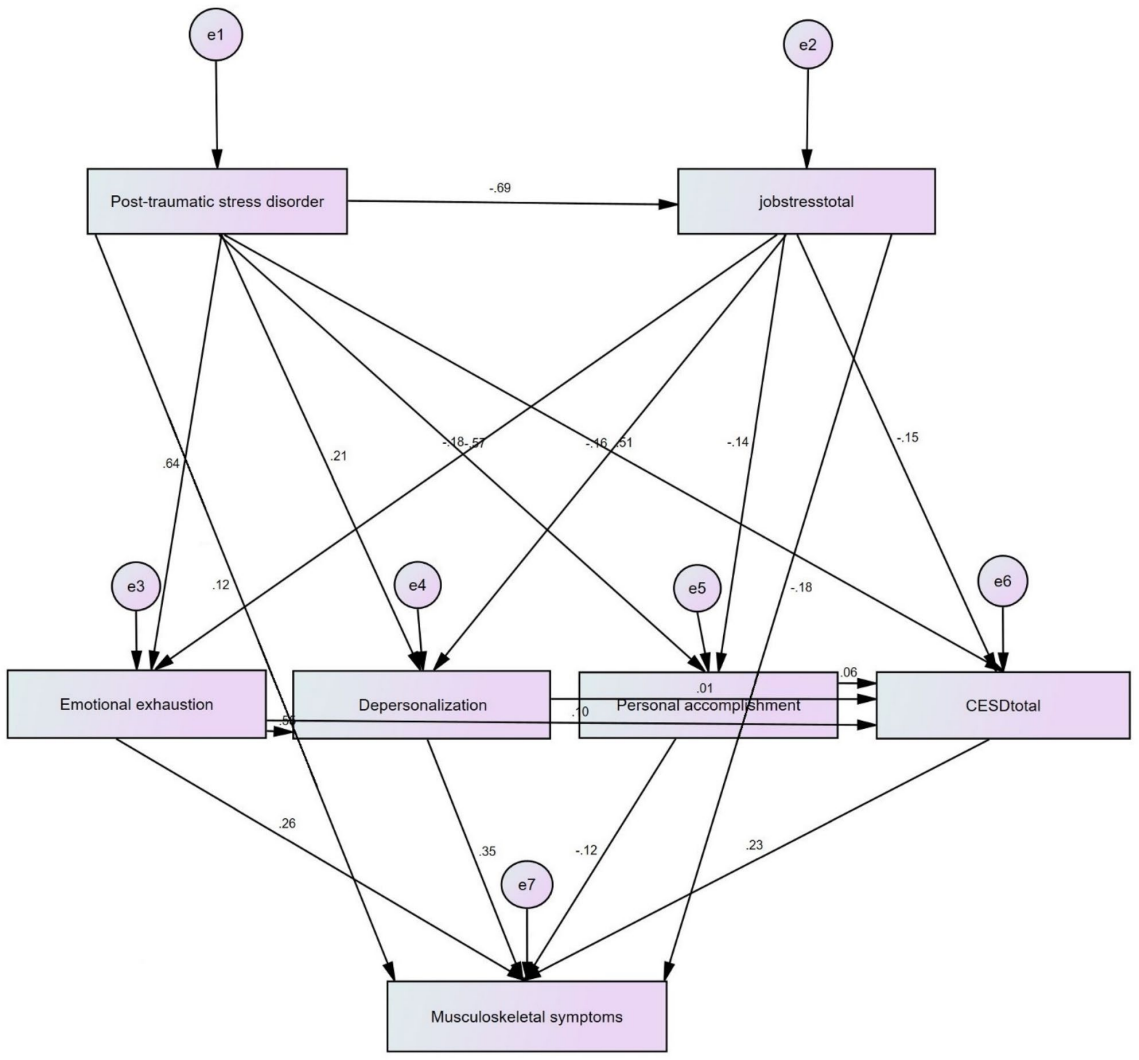
Model designed for examination of the relations between the studied variables.

The study examines the relations among the studied variables using the model presented in Fig. 2. The arrows indicate the relationship direction between two variables, and the values on the arrows show their direct effect coefficients.

Table 4 reports the effect coefficients of the variables on musculoskeletal symptoms. The results suggest that PTSD can affect musculoskeletal symptoms through ten paths. The highest effect coefficient of PTSD on musculoskeletal symptoms was indirectly through emotional exhaustion (0.166). Based on the results, job stress can impress on musculoskeletal symptoms through five paths. The greatest effect coefficient of job stress on musculoskeletal symptoms was direct (0.176). Among burnout dimensions, depersonalization had the highest effect coefficient on musculoskeletal symptoms (0.350). The direct, indirect, and total effect coefficients of the studied variables on musculoskeletal symptoms are shown in Table 5. The results revealed that the highest direct effect coefficient was related to depersonalization (0.350) and greatest indirect and total effect coefficients belonged to PTSD (0.653 and 0.777), respectively. As a result, the indirect effect coefficient of PTSD on musculoskeletal symptoms was higher of direct effect coefficients, which show the mediating role of the studied variables. Table 6 represents the goodness-of-fit indices of the model. The results demonstrated that the fitness of the model was confirmed.

**Table 4:** The effect coefficients of the PTSD on WRMSDs.

Variable	Effect coefficient
PTSD → Musculoskeletal symptoms	0.124
Job stress → Musculoskeletal symptoms	0.176
PTSD → Job stress → Musculoskeletal symptoms	0.121
PTSD → Job stress → Emotional exhaustion → Musculoskeletal symptoms	0.032
PTSD → Job stress → Depersonalization → Musculoskeletal symptoms	0.039
PTSD → Job stress → Personal accomplishment → Musculoskeletal symptoms	0.012
PTSD → Job stress → Depression → Musculoskeletal symptoms	0.024
PTSD → Emotional exhaustion → Musculoskeletal symptoms	0.166
PTSD → Depersonalization → Musculoskeletal symptoms	0.074
PTSD → Personal accomplishment → Musculoskeletal symptoms	0.068
PTSD → Depression → Musculoskeletal symptoms	0.117
Job stress → Emotional exhaustion → Musculoskeletal symptoms	0.047
Job stress → Depersonalization → Musculoskeletal symptoms	0.053
Job stress → Personal accomplishment → Musculoskeletal symptoms	0.017
Job stress → Depression → Musculoskeletal symptoms	0.035
Emotional exhaustion → Musculoskeletal symptoms	0.260
Depersonalization → Musculoskeletal symptoms	0.350
Personal accomplishment → Musculoskeletal symptoms	- 0.120
Depression → Musculoskeletal symptoms	0.230
Emotional exhaustion → Depression → Musculoskeletal symptoms	0.023
Depersonalization → Depression → Musculoskeletal symptoms	0.002
Personal accomplishment → Depression → Musculoskeletal symptoms	0.014

**Table 5:** The direct, indirect, and total effect coefficients of the studied variables on musculoskeletal symptoms.

Variables	Direct effect	*P* value	Indirect effect	*P* value	Total
Emotional exhaustion	0.260	*P* < 0.001	0.023	*P* > 0.05	0.283
Depersonalization	0.350	*P* < 0.001	0.002	*P* > 0.05	0.352
Personal accomplishment	− 0.120	*P* < 0.001	0.014	*P* > 0.05	− 0.106
Depression	0.230	*P* < 0.001	–	–	0.230
PTSD	0.124	*P* < 0.001	0.653	*P* < 0.001	0.777
Job stress	0.176	*P* < 0.001	0.152	*P* < 0.001	0.328

**Table 6:** The fit indices of the model.

Indices	Name	Fitness	Obtained value
Absolute fitness indices	Goodness-of-fit index (GFI)	> 0.9	0.943
	Adjusted goodness-of-fit index (AGFI)	> 0.9	0.929
Comparative fitness indices	Normed fit index (NFI)	> 0.9	0.913
	Comparative fit index (CFI)	> 0.9	0.961
	Incremental fit index (IFI)	0–1	0.964
Normed fit index	Root mean squared error of approximation (RMSEA)	< 0.08	0.055
	Normed Chi-square (X2/df)	1–3	1.770

## Discussion

The aim of this study was to examine the relationship between occupational stress and WRMSDs in firefighters. This study also examined the mediating role of depression and job burnout on these relationships. As will be discussed, PTSD has an indirect effect on musculoskeletal symptoms through job stress, burnout dimensions, and depression was proven. We also examined that occupational stress would influence burnout dimensions and depression and through these factors affect musculoskeletal symptoms indirectly was also proven.

Occupational stress is considered a major cause of many undesired workplace outcomes, including WRMSDs^43^. Due to the demanding nature of their tasks, which include activities like running, climbing, dragging, and lifting, firefighters often encounter elevated levels of work-related stress^44,45^. The elevated work-related stress of firefighters is found to increase their risk of WRMSDs^44,45^.

The analysis undertaken as part of this study suggests that occupational stress is linked to WRMSDs through depression and burnout dimensions (Emotional exhaustion, depersonalization, and personal accomplishment). The findings are supported by the wider literature. In a study of 1239 nurses, nurses who felt depressed at the start of the study were more likely to report experiencing neck or shoulder pain 13 months later^46^. A physiological mechanism for this relationship posits that stress, can trigger depression and further the risk of muscle tension and pain, causes changes in blood flow and oxygen supply, and increases the production of algesic substances in the muscles; especially for people with prolonged (chronic) muscle pain^47^. The relationship between psychosocial factors, depression, and musculoskeletal disorders has been confirmed by previous studies in which chronic pain and depression often co-occur, meaning that people who suffer from pain have an increased risk of developing depression and people who suffer from depression experience pain^47^. Our findings add some degree of clarity to this cyclic relationship literature and suggest that depression offers a motivational mechanism between stress and WRMSDs.

Among burnout dimensions, depersonalization was found to be a strong mediator between stress and WRMSDs. Depersonalization is viewed as an adaptive response to emotional exhaustion^41^. Despite the influence of various factors on depersonalization, it is important to pay attention to depersonalization due to its mediating role in the relationship with WRMSDs. The connection between depersonalization and burnout is underscored by Yong et al.^48^, who identified a depersonalization score equal to or exceeding 11 as indicative of severe burnout. In their study of 1325 coal miners, 84.7% of the miners who suffered a WRMSD were classified as suffering from severe burnout (mean depersonalization score was 12:61 ± 6:90 points). Therefore, although job burnout poses a risk for WRMSD, its relationship is shaped by depersonalization^48^. This observation is clarified by psychosocial factors, which are recognized to profoundly affect WRMSD^48,49^ and was confirmed in this study, whereby it was through depersonalization, that job stress had the most significant impact on musculoskeletal symptoms (coefficient = 0.053; See Table 4). Given that job stress is a major cause of WRMSDs^51^ and firefighters have demanding jobs facilitating elevated levels of work-related stress^26,52^, this finding between job stress and WMSD is not unexpected.

Findings of this study highlight the significant correlations between job stress and both control and peer support (0.79 and 0.81 respectively, see Table 3). These findings are supported by previous literature which has found that low control over work and lack of social support from colleagues has been found to be related to musculoskeletal diseases^26^, as has low social support in the workplace and low satisfaction in employees^26^. Consistent with previous literature^50,51^, role conflict was correlated with measures of psychological distress and, in particular, job stress (0.75, see Table 3). This supports the idea that conflicting demands from multiple sources (e.g., multiple supervisors) may have a significant effect on workers' psychological well-being and the supposition that role conflict induced psychological states are associated with increased WRMSD complaints^52^. In conjunction with previous research, the findings of this study are consistent with those of Sauter and Swanson^53^, who proposed that psychosocial job stressors lead to higher reports of WRMSD through their impacts on psychological stress. Furthermore, the findings of this study align with prior research that emphasizes the significance of psychological distress in understanding how job-related role conflict can contribute to diminished physical health^54^. In this study, the demands of the job exhibited significant impact coefficients among the various dimensions of job stress (0.77: see Table 3). This is supported in other occupations where jobs with high demand are likely to put more strain on job incumbents and lead to low worker physical and mental wellbeing^52,55^.

Furthermore, the study revealed a positive and significant association with work-related musculoskeletal disorders (WRMSDs). Post-traumatic stress symptoms (PTSS) can arise following exposure to traumatic events such as violence, disasters, severe accidents, and injuries^56,57^. The findings of this study confirmed serial mediation between PTSD and WRMSDs through occupational stress and depression and occupational stress and burnout. The results of this study (See Table 4) showed that PTSD can affect musculoskeletal symptoms via eight paths (For example: PTSD → Occupational Stress → Depression → Musculoskeletal Symptoms). The highest effect coefficient of PTSD on musculoskeletal symptoms was through depersonalization (0.141). This finding corroborates with extant literature that suggests that PTSD is significantly related with WRMSDs symptoms such as pain, ache, and strain^56^. Although empirical evidence suggests a significant association, most of this evidence is from other contexts such as war veterans^57^, nursing etc.^24^. In military personnel, PTSD has been associated with personnel experiencing MSDs symptoms^56^. Findings suggest that PTSD is a potential trigger for chronic pain^58^. Thus, a two-way relationship between PTSD and pain may exist and WRMSD may be associated with PTSD as cause or effect. Thus, the results of this study suggest the association of psychological factors with WRMSDs.

### Theoretical implications

Findings of this paper have potential to contribute both theoretical and in practices. Majority of extant research in WRMSDs has focused on risk factors such as ergonomics (body posture, body vibration, and awkward positions)^59^, manual material handling^60^, previous pain episodes or history, age, body mass index, work experience, duration of work shift^61^, and so on. The role of psychological risk factors in WRMSDs has received less attention. Addressing the gap in the literature, the present study investigated and confirmed the associations between work-related stress and WRMSDs. Thus, this study contributes and extends the extant WRMSDs literature highlighting that psychological factors are also important from the perspective of their association with WRMSDs. Further, the findings from this study confirm the legitimacy of a job demand control model and substantiate the strain hypothesis in the WRMSDs context whereby jobs with higher demands are likely to place more strain on job incumbents and lead to low physical and mental wellbeing. Second, our findings address how these factors affect the stress and WRMSDs relationships and reveal the existence of mediation and serial mediation of depression and burnout. The research explains the mediation mechanism of burnout and depression between occupational stress and WRMSDs relationship and answers the question around how psychological factors are associated with WRMSDs. Third, by studying the firefighter’s data from an Iranian context, this paper adds to the theoretical legitimacy of the WRMSDs and their triggers. It is evident that the majority of study on WRMSDs are from either developed economies or from traditional business sectors such as manufacturing sector etc. and the public utilities/services sector are understudied. Hence, examining firefighters’ data would extend our understanding of the phenomenon.

### Practical implications

With a comprehensive understanding that job stress, along with mediating factors, contributes to work-related musculoskeletal disorders (WRMSDs), organizations can implement informed interventions to assist firefighters in reducing their stress as a strategy to address WRMSDs. For example: fire service (and other) organizations may consider shortening the work shift of firefighters who are deployed to more demanding stations or rotate firefighters among high demand to low or moderate demand station assignments periodically. Stations might also include fun and social activities to help mitigate firefighter job stress and depersonalization. Finally, firefighters with symptoms of depression, burnout, or PTSD should be provided with professional counselling services as soon as symptoms become evident.

### Study limitations

While extending knowledge in job stress and WRMSDs, generalization of these findings should be made with caution given this study’s limitations. Firstly, the study's design was cross-sectional, and since we utilized the same data source, we cannot completely rule out the potential for common method bias (CMB). Although we have taken various measures to take care of CMB, we suggest prospective cohort studies to overcome this limitation. Secondly, the scope of the study was limited to a few psychological factors and a replication of this research with more psychological risk factors is advisable. Third, since the majority of factors are individual related, it would be interesting to consider certain factors as control variables in future research. Finally, the research was within a firefighting population, an occupation which has unique and highly challenging requirements. We suggest more such types of examination be conducted in different work setups and professions to generalize the findings.

## Conclusion

Objective of this paper is to examine the relationship between occupational stress and WRMSDs among firefighter workers in an Iranian context. In addition, we tested the mediation of depression and burnout between job stress and WRMSDs. To achieve these objectives, a sample set of 2339 Iranian firefighters were collected using survey questionnaire. The collected data was analyzed using structural equation modelling. Findings of our examination suggest that occupational stress among firefighters leads to work related musculoskeletal disorders. Also, we found that depression and burnout dimensions (emotional exhaustion, depersonalization, and personal accomplishment) mediate the relationship between occupational stress and WRMSDs. Our findings also suggest presence of serial mediation between PTSD and WRMSDs via stress and depression, and stress and burnout dimensions. To conclude, our study suggests that psychological factors are related positively with WRMSDs of firefighters. On the basis of our examination, we recommend that organizations should design interventions to prevent and manage occupational stress, PTSD, burnout, and depression so that their undesirable consequences (WRMSDs in present case) can be controlled.

WRMSDs are identified by WHO-ILO reports and other reports such as EU-OSHA reports as one of the top three factors that affect productivity and performance of organizations, sectors and economies. Knowing what causes WRMSDs and what interventions can help in managing this undesired evil at workplace would help organizations and policy makers in designing appropriate interventions.

## Data Availability

The datasets used and/or analyzed during the current study are available from the corresponding author upon reasonable request.
